# Assessing Metal Exposure and Leaching from Discarded Cigarette Butts: Environmental Analysis and Integrated Waste Management Approaches

**DOI:** 10.3390/toxics12050324

**Published:** 2024-04-29

**Authors:** Muhammad Faisal, Zai-Jin You, Noman Ali Buttar, Muhammad Bilal Idrees, Muhammad Naeem, Shoaib Ali, Basharat Ali, Abeer Hashem, Elsayed Fathi Abd_Allah

**Affiliations:** 1 Centre for Ports and Maritime Safety, Dalian Maritime University, Dalian 116026, China; b.you@dlmu.edu.cn; 2 Department of Agricultural Engineering, Khwaja Fareed University of Engineering and Information Technology, Rahim Yar Khan 64200, Pakistan; dr.basharat@kfueit.edu.pk; 3 Faculty of Infrastructure Engineering, Dalian University of Technology, Dalian 116024, China; bilal@dlut.edu.cn; 4 Key Laboratory of Water Cycle and Related Land Surface Processes, Institute of Geographic Sciences and Natural Resources Research, Chinese Academy of Sciences, Beijing 100101, China; mnaeem2022@igsnrr.ac.cn; 5 Department of Earth and Space Sciences, Southern University of Science and Technology, Shenzhen 518005, China; shoaib@sustech.edu.cn; 6 Botany and Microbiology Department, College of Science, King Saud University, P.O. Box. 2460, Riyadh 11451, Saudi Arabia; habeer@ksu.edu.sa; 7 Plant Production Department, College of Food and Agricultural Sciences, King Saud University, P.O. Box. 2460, Riyadh 11451, Saudi Arabia; eabdallah@ksu.edu.sa

**Keywords:** cigarette butts, metals, hazardous waste, waste management, littered waste

## Abstract

Cigarette butts, often discarded as litter, are considered a common form of waste, containing a variety of pollutants within this hazardous residue. This study, which was designed to assess the environmental release of certain metals from cigarette butts, investigates a variety of scenarios under varying climatic conditions. Thus, in order to assess the level of metal contamination, samples of cigarette butts were collected in urban areas from seven popular brands in China, smoked artificially, and examined through graphite furnace atomic absorption (GF-AAS). The findings indicated mean concentrations of 1.77 for Cr, 2.88 for Ni, 12.93 for Cu, 24.25 for Zn, and 1.77 µg/g for Pb in the case of newly smoked butts. The emission of each of the metals increases to 8–10% when cigarette butts remain in the environment for an extended period of time. Furthermore, rainfall can accelerate metal leaching, reaching values of 18–20% compared to the controlled scenario. The worst-case scenario releases 2129.31 kg/year of metals into the environment, while the best-case scenario sees a lower release of 844.97 kg/year. The data reflect variations in metal emissions across different scenarios. There was also a strong correlation between cigarette butts in public spaces and cities. This research highlights the need to educate smokers and increase urban maintenance efficiency to reduce this litter and the metals it leaches into the environment.

## 1. Introduction

Cigarette butt contamination poses a significant challenge to urban waste management [1,2]. Today, cigarette usage is dominant in several countries, especially among youth [3,4]. In some countries, this number has grown to several hundred billion cigarettes a year [5,6]. Over the past 70 years, the popularity of filtered cigarettes has led to the creation of a new type of waste consisting of filter leftovers, wrappings, and unused tobacco [4]. Even though they are small, cigarette butts are a major source of litter around the world, and users often do not care about them, causing problems with accumulation [7,8]. Waste collection agencies are facing significant challenges due to the widespread distribution of cigarette butts caused by smokers. Cigarette butts are considered long-lasting garbage in urban and public areas; collecting them is expensive because the collection equipment is ineffective and the butts are small [9]. Problems related to cigarette butts include their large quantities, their possible widespread dispersion, and the existence of various toxins within the butts that may periodically leach into the environment. Cigarette butts contain thousands of compounds, hundreds of which are known to cause cancer and genetic mutations. Consequently, potential harm to the ecosystem and other forms of life is a major issue with cigarette butts [10]. Cigarette butts have been found to contain significant amounts of metals, including lead, cadmium, copper, nickel, zinc, and chromium [11]. Cigarette butts include a wide variety of contaminants, from these metals to organic chemicals to PAHs to nicotine [12]. These harmful contaminants escape from abandoned cigarette butts and contaminate the ecosystem [13,14,15]. Cigarette butt poisonous contents infiltrate into both soil and water [16,17]. Cigarette butt leakage can contaminate water sources, raise the concentration of contaminants in waste from municipal dumps, and produce leachate [18]. Other potential effects of littering with cigarette butts include the risk of fire and the potential for animals and young children to choke on the butts [19]. The reduction in cigarette butt pollution necessitates an approach that incorporates several different strategies, including infrastructure improvement, community involvement, regulatory enforcement, and public education. These types of initiatives are critical to encouraging appropriate disposal methods and reducing harm to the environment. There is a big gap in the research on measuring metal emissions and comparing different ways to handle them. Filling this gap is critical for making effective policy decisions and reducing the global environmental impact of cigarette butt waste. This research had the following objectives: (1) to estimate the metal concentration from different brands of littered and newly smoked cigarette butts; and (2) to evaluate various scenarios in order to determine the total quantity of metals released by cigarette butts. Several strong factors make this research highly relevant to an international audience. It will assess the environmental impact of littered and recently smoked cigarette butts. Examining various scenarios to determine the metal emissions from cigarette butts illuminates the extent of metal pollution this waste type generates, which offers a valuable insight for global policymakers, environmental organizations, and public health officials. This study highlights the importance of implementing appropriate mitigation methods and public awareness campaigns worldwide by revealing the full level of metal contamination in cigarette butt litter. It also adds to our scientific knowledge of environmental pollution.

## 2. Materials and Methods

### 2.1. Study Area

**Figure 1:**
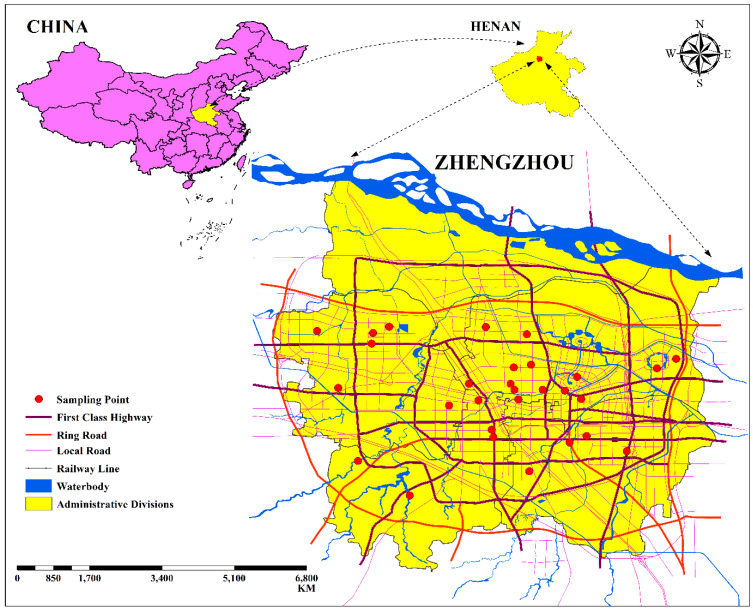
Study area map.

This study is focused on Zhengzhou city, the capital of Henan Province, located (Figure 1) at (34°45′50.4″ N, 113°41′2.4″ E) [20,21]. Positioned as a significant economic and administrative hub in the middle of China, Zhengzhou plays a crucial role in the vast Central Belt metropolis of the province [22,23]. Zhengzhou, nestled at the foothills of the Funiu Hills in the far north, shares a total geographical area of 1011.3 km^2^ with upland regions to the west and modest and lowland terrain to the east. The climate in Zhengzhou is characterized by an average annual precipitation of 629.7 mm, with the majority of rainfall occurring during the summer. Rapid economic growth and urban expansion have transformed Zhengzhou into a swiftly expanding metropolis in the central region of China. However, this development has come at a cost, notably impacting the city’s air quality, which has become a pressing concern due to the city’s burgeoning urbanization and economic activities [24,25].

### 2.2. Sampling Process

For this particular research project, we selected seven distinct brands (B1, B2, B3, B4, B5, B6, and B7) of cigarettes to initiate the sampling procedure.

For “newly smoked”: We employed a special device, a pump which consists of a mechanism that generates airflow to puff on the cigarettes at a consistent rate and intensity. The pump device is equipped with adjustable settings to regulate factors such as puff duration, puff volume, and puff frequency, ensuring reproducibility and accuracy in the experimental setup, to puff on the cigarettes in an orderly manner. Our goal was to keep the smoking conditions consistent throughout the entire experiment. After smoking, we carefully measured and handled the leftover cigarette butts, getting them ready for the next steps in our research.

“Littered cigarette butts” are the ones we gathered from city areas. We have taken the time to randomly collect at least 200 pieces of each sample from locations along the roadside. This meticulous collection process ensures a diverse and representative set of cigarette butts for our study. We have strategically selected two distinct time categories for the collection of littered cigarette butts to introduce temporal variability into our research. The first category involves a temporal window of 48 h post winter rainfall conditions, while the second category spans a timeframe of 15 days characterized by abundant sunshine in the summer season [26].

### 2.3. Laboratory Analysis

Once the sample collection process concluded, we swiftly transported all samples to the laboratory, ensuring their storage in a cool and dry environment. We set up a 24 h window to fully analyze these samples and ensure the integrity of the gathered data. We simulated rain by using a shower at 10 mm per hour and placing fresh cigarette butts on top of the sediments. The duration of this simulated precipitation was two hours. We performed this in order to observe the effects that it had on the cigarette butts. To ensure the reliability of our data, it was critical to carefully move and set up the experiment [3]. To simplify this estimation, a solution consisting of 3M nitric acid (HNO_3_) and 2M hydrochloric acid (HCl) in a ratio of 1:3 was utilized. We examined the metal levels in the filtered samples using graphite furnace atomic absorption (GF-AAS). We utilized a total of 5 different scenarios and 6 different situations, which are detailed in Table 1 and Table 2, to estimate the possible metal releases from cigarette butts. We used these hypothetical situations to estimate the potential metal releases from cigarette butts.

Each scenario represents assumptions about how long cigarette butts reside in urban environments. In addition, we aimed to estimate the yearly metal emission from cigarette butts by utilizing data obtained from experiments using various types of cigarette butts. 

To guarantee the precision and dependability of our examination of the elements chromium (Cr), copper (Cu), zinc (Zn), lead (Pb), and nickel (Ni) in cigarette butts taken from samples of road dust, we carefully followed quality control procedures in our experiment. In order to evaluate background signals and possible contamination, we included blank samples in each batch that were devoid of the analytes of interest for blank analyses. The preparation and analytical processes for these blank samples were the same as those for the real samples. For the recovery experiments, we spiked representative samples with known amounts of Cr, Cu, Zn, Pb, and Ni at a rate of 10 µg/g. We computed the recovery percentages by comparing the measured concentrations of the spike analytes to their known concentrations. The average recovery percentages for Cr, Cu, Zn, Pb, and Ni were 98%, 96%, 97%, 96%, and 94%, respectively, demonstrating the effectiveness of our sample preparation and extraction processes. The quantification limits (LOD and LOQ), and CRM certified concentrations were as follows (Table 3).

## 3. Results and Discussions

**Figure 2:**
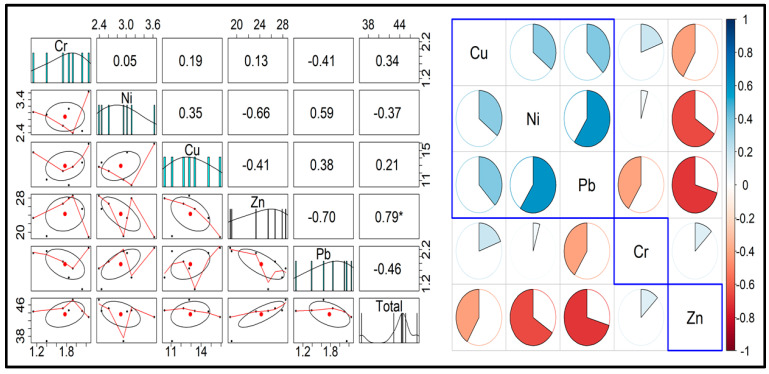
Statistical plots for metal concentration in seven different brands of cigarettes. (* represent a significance level of *p* < 0.05).

The study compiled and easily accessed the results of determining the concentrations of five different metals in the cigarette butts connected to seven popular high-consumption cigarette brands (N = 200 per sample) available in the Chinese market, as shown in Table 3. Statistical analysis values are presented in Table 4 and Figure 2, where correlation and pair-panel plots are shown. 

In this table, we can see a breakdown of the elements of seven distinct brands, with an emphasis on Cr, Ni, Cu, Zn, and Pb. B3 stands out with the highest Ni content at 3.63 µg/g, while B4 has the lowest Cr content at 1.13 µg/g. Cu content varies considerably, with B3 leading at 15.89 µg/g and B2 trailing at 10.36 µg/g. Zn content is highest in B7 at 28.5 µg/g and lowest in B3 at 18.85 µg/g. Additionally, B2 exhibits the highest Pb content at 1.39 µg/g, while B1 has the lowest at 1.05 µg/g. Concerns about the possible effects on human and environmental health are driving interest in the metal concentrations, especially those of Ni, Cu, and Pb. At 3.63 µg/g for Ni and 15.89 µg/g for Cu, B3 possesses the highest concentrations of these elements. Given the environmental impacts and potential health risks, further investigation into elevated levels of specific metals may be necessary. On the other hand, Pb concentrations across all brands are generally low, with B2 recording the highest at 1.39 µg/g. Any lead presence is concerning, so it is important to keep an eye on these numbers even though they are lower than usual health recommendations. All brands’ elemental composition can be measured against these averages. Cr and Ni have mean values of 1.77 µg/g and 2.88 µg/g, respectively, suggesting a relatively consistent presence across the brands. Cu exhibits a higher mean at 12.93 µg/g, indicating a generally elevated copper content in the dataset. Zn and Pb also show higher mean values at 24.25 µg/g and 1.77 µg/g, respectively, underscoring the importance of considering these elements collectively in any assessment. Looking at the highest and minimum values reveals the range of variability, providing a better understanding of the dataset’s extremes. Cr varies from 1.13 µg/g to 2.25 µg/g, Ni from 2.39 µg/g to 3.63 µg/g, Cu from 10.36 µg/g to 15.89 µg/g, Zn from 18.85 µg/g to 28.5 µg/g, and Pb from 1.05 µg/g to 2.25 µg/g. Because different cigarette brands have different elemental compositions, it is important to conduct individualized evaluations based on specific application requirements or government regulations. While metal analysis raises health and environmental issues, distinct patterns reveal individual brands’ strengths and weaknesses. One way to evaluate dataset diversity is to use variability ranges, while mean values can provide a central reference. With this summary, industry and regulators may better understand product composition, make decisions about quality control, and manage the environment. The results show that even when exposed to the same environmental circumstances, cigarette butt concentrations can vary significantly across brands.

Furthermore, Table 5 demonstrates the widely recognized large variations in the metal content of samples collected under varied environmental conditions. We found that the metal content was lowest under long-persistent and wet conditions, and highest under short-persistent and bright sunlight.

**Figure 3:**
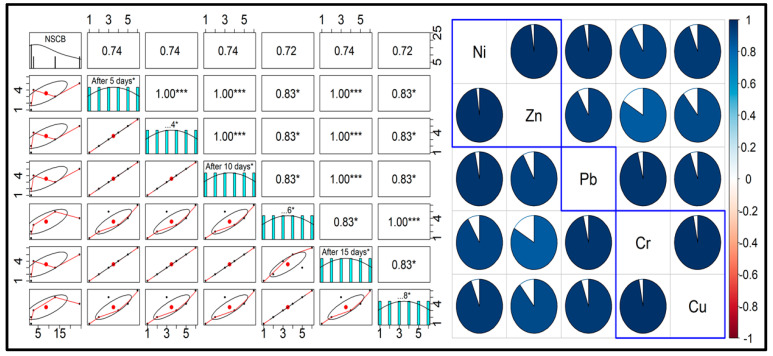
Statistical plots for metal concentration in newly smoked cigarette butts under different conditions and days. (* represent a significance level of *p* < 0.05. *** represent a significance level of *p* < 0.001, and red dots are used to highlight significant correlations visually).

Across the 15-day timeframe, Cr, Ni, and Cu consistently displayed declining concentrations, indicative of potential chemical transformations or leaching mechanisms at play. Notably, rainy conditions appeared to amplify this reduction, suggesting a stronger environmental influence during precipitation events. This suggests a different response to temporal aging or environmental influences, highlighting the complexity of metal behavior in cigarette litter. Pb concentrations, in contrast, displayed noticeable fluctuations. The fluctuating pattern was evident under both sunny and rainy conditions, emphasizing the intricate interactions between atmospheric components and this particular metal. The statistical analysis for Table 5 is given in Figure 3. The temporal trends collectively underscore a general reduction in metal concentrations over time, aligning with expectations of potential leaching or alterations within the discarded cigarette butts. Rainfall’s influence, particularly in accelerating the decline of certain metals, adds an environmental dimension to the observed dynamics. In summary, the nuanced temporal and environmental variations in metal concentrations within discarded cigarette butts emphasize the need for a comprehensive understanding of both aging processes and external factors in waste management considerations. The intricate nature of these interactions underscores the importance of developing effective strategies for mitigating environmental and public health risks associated with discarded cigarette litter. The concentrations of metals in littered cigarette butts are given below in Table 6. Comparing the two tables, the concentrations of metals exhibit variations in response to different environmental conditions and temporal intervals. In Table 5, metal concentrations after 15 days generally displayed a decreasing trend, with slight fluctuations.

Contrarily, Table 6, reflecting conditions after 15 sunny days and 48 h of rainy weather, unveils nuanced patterns. In this table, concentrations often align with or slightly exceed previous levels, indicating potential influences of seasonal changes and pollution levels. Cr, Ni, and Zn concentrations, in particular, show distinctive trends in response to varying conditions. These comparisons underscore the dynamic nature of metal behavior, emphasizing the importance of considering both temporal evolution and environmental factors for a comprehensive understanding in environmental studies.

**Figure 4:**
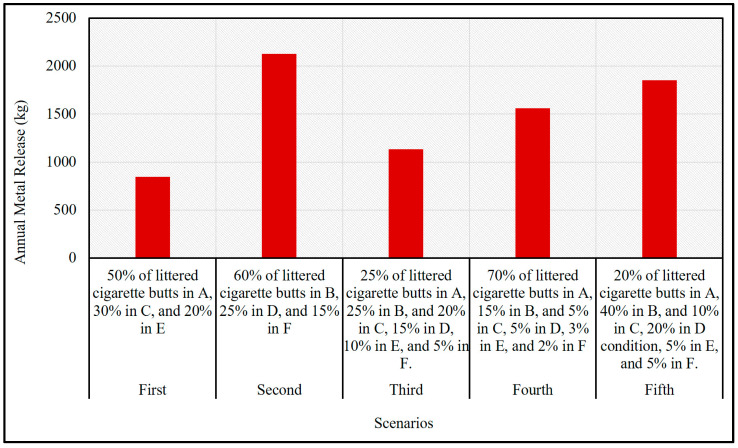
Scenario-based annual release of metals from littered cigarette butts.

The research concluded that cigarette butts, which contain high levels of metals and many other toxins and poisons, are hazardous waste products that can pollute water and kill species [3,10,27]. Because they contain a lot of metals, cigarette butts are considered hazardous waste [10,27]. Additionally, cigarette butts can harm plant growth if they are in the air [28]. Cigarette butts produce this toxic material by releasing chemicals into the environment [15]. Humid environments facilitate the release of tobacco filter pollutants more readily. In damp environments, cigarette butts exhibit lower metal concentrations as a result of increased leaking. Despite the fact that cigarette butts do not change significantly over time, they do release pollutants at different rates, which leads to a decrease in the amount of metal that is present in the environment. Examining cigarette butts for metals proves that they are a known metal-release source, but determining precise levels due to environmental variables and structural variations in cigarettes is difficult. Metal discharges from cigarette butts in the Chinese environment can vary between 844.97 kg and 2129.31 kg per year, according to the scenarios evaluated in the present research (Figure 4).

These estimates are based on the consumption of 2.4 trillion cigarettes in China every year, assuming that 80 percent of the cigarette butts produced end up in landfills. Even when we dispose of cigarette butts through the proper channels, there remains a potential for the indirect release of metals into the environment. This is an extremely important fact to acknowledge. Because cigarette butts are present in dumped urban garbage, the concentration of such metals in drainage is significantly higher. The discharge of contaminants like metals from cigarette butts poses a concern for public health since these metals have negative impacts on human health and spread to soil and water sources, increasing the likelihood of sickness in society [16,29]. 

**Figure 5:**
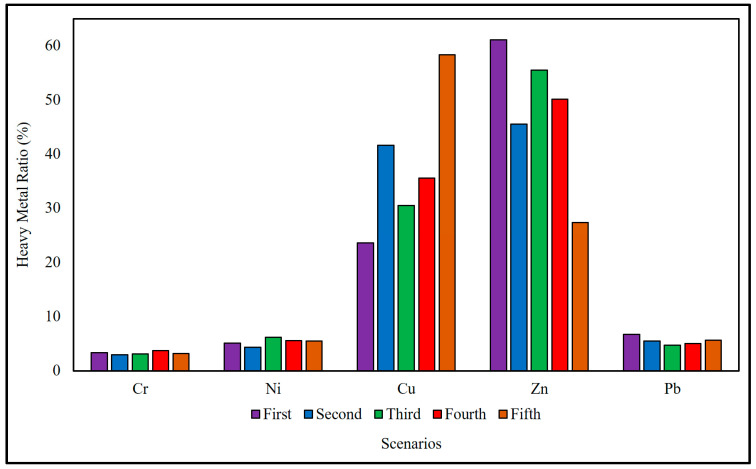
Metal leakage ratio on the basis of scenarios.

Figure 5 presents a comprehensive overview of the metal concentration ratio across five distinct scenarios. Each scenario represents a specific set of conditions or factors that influence the concentration levels of these metals. In the first scenario, Cr stands out with a concentration ratio of 3.38%, while Zn dominates with an exceptionally high concentration ratio of 61.13%. The second scenario exhibits a lower concentration ratio of Cr and Ni but a substantial increase in Cu and Zn, indicating a shift in the distribution of these metals. The third scenario introduces a moderate increase in Cr and Ni concentration ratios, with a slight decrease in Cu and Zn. Interestingly, Pb experiences a significant decline. Moving to the fourth scenario, there is a slight increase in the Cr and Cu concentration ratios, while Ni and Zn maintain relatively stable levels. Pb also sees a minor increase. The fifth scenario presents a significant shift in metal concentration ratios, particularly in Cu and Zn, with values soaring to 58.32% and 27.32%, respectively. Cr and Ni exhibit marginal increases, while Pb remains relatively stable. Comparing the scenarios reveals intriguing patterns in the distribution of metals. Notably, Zn appears to be a pivotal metal, displaying substantial variations across the scenarios. Scenario three shows a balance in metal concentrations, while scenario five accentuates the dominance of Cu and Zn. Cr and Ni, although displaying some fluctuations, maintain relatively consistent levels. Pb, on the other hand, demonstrates subtle changes, reflecting the scenario-specific dynamics. This comprehensive analysis underscores the complexity of metal distribution and highlights the importance of considering various scenarios and their unique environmental conditions. Understanding these variations is crucial for developing effective environmental management strategies, especially in contexts where metal contamination poses potential risks to ecosystems and public health. For this kind of observation, the impact of cigarette butts that are present in various ecosystems is a key consideration. Because most cigarette butts remain in place until the next day’s clean-up and weather conditions vary from day to day, the amount of metal that leaks out of them varies in urban contexts [7]. Contaminating a wide variety of organisms, cigarette butts deplete water and soil resources and pose a serious threat to human health. There are significant concerns over the possibility of cigarette butt contamination [3]. Factors such as the effectiveness of municipal amenities and maintenance, as well as the existence of areas with limited accessibility, affect the overall duration that scattered cigarette butts persist in the environment [7]. Some claim that different parts of Madrid have varying cleaning standards, leading to a higher concentration of littered cigarette butts in certain regions due to their prolonged persistence [30]. According to the study’s authors, cigarette butts absorb more metal when stored in inconvenient places like water ditches and bike racks. It is not possible to completely remedy the problem by simply collecting butts or appropriately disposing of them, because doing so may result in the discharge of toxins. We must reduce the generation of butts through cessation programs, provide education to promote safe disposal, and make technological developments to decrease contaminants. This requires a multidisciplinary approach. The development of eco-friendly alternatives and the implementation of pre-landfill extraction procedures achieve the goal of minimizing environmental harm and reducing pollutants in landfills. Controlling the leakage of contaminants, including metals, from cigarette butts is another vital aspect of environmental protection. Improved waste management systems, coupled with stringent regulations, can help prevent the escape of harmful substances from discarded cigarette butts, safeguarding the surrounding environment.

Lastly, establishing designated areas for the disposal of cigarette butts can contribute to better waste management practices. By implementing regulations that confine the littering of cigarette butts to specific locations, authorities can exert greater control over the environmental impact of these pollutants, ensuring proper containment and disposal. In conclusion, addressing the environmental challenges posed by cigarette butts requires a holistic approach encompassing reduced generation, responsible disposal, pollutant reduction, advanced waste management, and regulatory measures. Only through a concerted effort in these areas can we hope to mitigate the adverse effects of cigarettes on our environment.

We accept various limitations in our models for calculating metal concentrations from cigarette butts and evaluating overall metal emissions, considering the goals stated and the data supplied. The heterogeneity in sampling, which results from different locations and collection techniques, casts doubt on the data’s representativeness. Because analytical techniques have inherent limitations, calibrating efforts may not always produce precise results. Environmental factors such as weather and pollution sources hampered accurately calculating metal deposition and redistribution. Human activity and degradation impact the temporal fluctuation in metal release rates, further complicating predictions. Our findings are further complicated by modeling assumptions regarding the behavior of littering. Furthermore, poor data quality and availability limit the model’s robustness. A refinement of techniques, sensitivity analysis, and validation against empirical data are necessary to address these shortcomings. We can enhance the model’s accuracy and deepen our understanding of the metal pollution stemming from cigarette butt litter by incorporating validation studies and field data. Despite these difficulties, our research offers insightful information about the effects on the ecosystem and emphasizes the necessity of sophisticated strategies for successfully reducing metal pollution.

## 4. Conclusions

This study has examined seven different brands and five different metals. With a Ni value of 3.63 µg/g, B3 is the most notable, whereas B4 has the lowest Cr content (1.13 µg/g). With B3 leading at 15.89 µg/g and B2 behind at 10.36 µg/g, the Cu content varies significantly. B7 has the highest zinc content (28.5 g/g), whereas B3 has the lowest (18.85 g/g). Furthermore, B2 has the greatest Pb concentration (1.39 µg/g) compared to B1’s lowest (1.05 µg/g). Concerns about potential impacts on human and environmental health, particularly those of Ni, Cu, and Pb, fuel interest in the metal concentrations. With concentrations of 3.63 µg/g for Ni and 15.89 µg/g for Cu, B3 has the highest concentration of these metals. Further research into elevated levels of specific metals may be necessary, given the environmental consequences and possible health risks. Conversely, Pb values are generally modest for all brands, with B2 having the highest concentration at 1.39 µg/g. Even though these values are lower than the standard health recommendations, any presence of lead is cause for concern, so it is crucial to monitor them. Any brand’s elemental content can be compared to this average. With mean levels of 1.77 µg/g and 2.88 µg/g, respectively, Cr and Ni appear to be present in most brands in a fairly consistent amount. Cu has a greater mean of 12.93 µg/g, suggesting that the dataset has a usually higher copper concentration. Higher mean values for zinc and lead (at 24.25 µg/g and 1.77 µg/g, respectively) further highlight the significance of taking these components into account in any assessment. Examining the greatest and lowest values improves our understanding of the dataset’s range of variability and its extremes. The ranges for Cr, Ni, Cu, Zn, and Pb are, 1.13 g/g to 2.25 g/g, 2.39 g/g to 3.63 g/g, 10.36 g/g to 15.89 g/g, and 18.85 g/g to 28.5 g/g, and 1.05 g/g to 2.25 g/g respectively. Because the elemental compositions of different brands of cigarettes vary, it is critical to carry out customized assessments based on application needs or legal restrictions. Metal analysis raises health and environmental concerns, while specific patterns highlight the advantages and disadvantages of particular brands. While mean values can serve as a core reference, variability ranges are one method of assessing dataset diversity. This overview can help industry and regulators make better decisions about quality control, environmental management, and product composition. The results demonstrate that cigarette butt concentrations can differ dramatically between brands even when exposed to identical environmental conditions. Metal leakage accelerates when cigarette butts remain in the atmosphere for extended periods and humidity levels rise. It was estimated that the annual emission of metals from cigarette butts into the Chinese environment is 844.97–2129.31 kg/year. Cigarette butts release contaminants like metals, which can have harmful effects on organisms. To mitigate this, there should be effective ways to clean and recycle cigarette butts, decrease littering by smokers, and decrease the amount of harmful substances in cigarette butts.

## Figures and Tables

**Table 1:** Established situations based on weather conditions.

Situations	Duration (Days)	Rainfall (mm/h)
A	5	0
B	5	10
C	10	0
D	10	10 (Three times)
E	15	0
F	15	10 (Four times)

**Table 2:** Established scenarios based on cigarette butts and situations.

Scenario	Terms and Conditions
First	50% of littered cigarette butts in A, 30% in C, and 20% in E
Second	60% of littered cigarette butts in B, 25% in D, and 15% in F
Third	25% of littered cigarette butts in A, 25% in B, 20% in C, 15% in D, 10% in E, and 5% in F
Fourth	70% of littered cigarette butts in A, 15% in B, 5% in C, 5% in D, 3% in E, and 2% in F
Fifth	20% of littered cigarette butts in A, 40% in B, 10% in C, 20% in D, 5% in E, and 5% in F

**Table 3:** Metal quantification limits (LOD and LOQ), and CRM certified concentrations.

Metals	LOD (µg/g)	LOQ (µg/g)	CRM Concentrations (µg/g)
Cr	0.1	0.3	10
Cu	0.05	0.1	20
Zn	0.02	0.05	30
Pb	0.1	0.3	15
Ni	0.02	0.05	25

**Table 4:** Metal concentration in seven different brands of littered cigarettes (µg/g).

Brands	Cr	Ni	Cu	Zn	Pb
B1	2.12	2.45	13.37	25.45	1.05
B2	1.85	3.12	10.36	27.89	1.39
B3	2.25	3.63	15.89	18.85	2.25
B4	1.13	3.02	14.74	23.33	2.1
B5	1.73	2.61	12.25	26.66	1.85
B6	1.4	2.94	11.11	19.1	2.15
B7	1.93	2.39	12.82	28.5	1.65
Mean	1.77	2.88	12.93	24.25	1.77
Maximum	2.25	3.63	15.9	28.5	2.25
Minimum	1.13	2.39	10.4	18.9	1.05
Standard Deviation	0.39	0.43	1.93	3.97	0.44
*p*-values	0.14	0.16	0.73	1.5	0.16

**Table 5:** Metal concentration in newly smoked cigarette butts under different conditions and days (µg/g).

Metals	Newly Smoked Cigarette Butts	After 5 Days		After 10 Days		After 15 Days	
		Sunny	Rainy	Sunny	Rainy	Sunny	Rainy
Cr	1.77	1.68	1.49	1.62	1.47	1.58	1.43
Ni	2.88	2.76	2.62	2.64	2.49	2.53	2.34
Cu	12.93	12.5	10.26	11.98	9.88	10.72	8.89
Zn	24.25	23.8	23.1	22.75	21.7	21.97	20.1
Pb	1.77	1.55	1.4	1.49	1.26	1.43	1.14
Mean	8.72	8.458	7.774	8.096	7.36	7.646	6.78
Minimum	1.77	1.55	1.4	1.49	1.26	1.43	1.14
Maximum	24.25	23.8	23.1	22.75	21.7	21.97	20.1

**Table 6:** Metal concentration in littered cigarette butts (µg/g).

Metals	After 15 Sunny Days (Summer)		After 48 h of Rainy Weather (Winter)	
	High-Pollution Area	Low-Pollution Area	High-Pollution Area	Low-Pollution Area
Cr	1.43	1.71	1.45	1.52
Ni	2.64	2.82	2.47	2.59
Cu	10.73	12.02	9.66	10.12
Zn	21.58	23.28	18.95	20.85
Pb	1.3	1.53	0.87	1.12
Mean	7.53	8.27	6.68	7.24
Minimum	1.3	1.53	0.87	1.12
Maximum	21.58	23.28	18.95	20.85

## Data Availability

The data presented in this study are available on request from the corresponding authors.

## References

[1] Yousefi M., Kermani M., Farzadkia M., Godini K., Torkashvand J. (2021). Challenges on the Recycling of Cigarette Butts. Environ. Sci. Pollut. Res..

[2] Chevalier Q., El Hadri H., Petitjean P., Bouhnik-Le Coz M., Reynaud S., Grassl B., Gigault J. (2018). Nano-Litter from Cigarette Butts: Environmental Implications and Urgent Consideration. Chemosphere.

[3] Roder Green A.L., Putschew A., Nehls T. (2014). Littered Cigarette Butts as a Source of Nicotine in Urban Waters. J. Hydrol..

[4] Araújo M.C.B., Costa M.F. (2019). A Critical Review of the Issue of Cigarette Butt Pollution in Coastal Environments. Environ. Res..

[5] Marah M., Novotny T.E. (2011). Geographic Patterns of Cigarette Butt Waste in the Urban Environment. Tob. Control.

[6] Aeslina A.K., Mohajerani A. Leachability of Heavy Metals from Fired Clay Bricks Incorporated with Cigarette Butts. Proceedings of the 2012 IEEE Symposium on Business, Engineering and Industrial Applications.

[7] Gholami M., Torkashvand J., Kalantari R.R., Godini K., Jafari A.J., Farzadkia M. (2020). Study of Littered Wastes in Different Urban Land-Uses: An 6 Environmental Status Assessment. J. Environ. Health Sci. Eng..

[8] Kataržytė M., Balčiūnas A., Haseler M., Sabaliauskaitė V., Lauciūtė L., Stepanova K., Nazzari C., Schernewski G. (2020). Cigarette Butts on Baltic Sea Beaches: Monitoring, Pollution and Mitigation Measures. Mar. Pollut. Bull..

[9] Rath J.M., Rubenstein R.A., Curry L.E., Shank S.E., Cartwright J.C. (2012). Cigarette Litter: Smokers’ Attitudes and Behaviors. Int. J. Environ. Res. Public Health.

[10] Parker T.T., Rayburn J. (2017). A Comparison of Electronic and Traditional Cigarette Butt Leachate on the Development of Xenopus Laevis Embryos. Toxicol. Rep..

[11] Ghasemi A., Golbini Mofrad M.M., Parseh I., Hassani G., Mohammadi H., Hayati R., Alinejad N. (2022). Cigarette Butts as a Super Challenge in Solid Waste Management: A Review of Current Knowledge. Environ. Sci. Pollut. Res. Int..

[12] Dobaradaran S., Schmidt T.C., Lorenzo-Parodi N., Kaziur-Cegla W., Jochmann M.A., Nabipour I., Lutze H.V., Telgheder U. (2020). Polycyclic Aromatic Hydrocarbons (PAHs) Leachates from Cigarette Butts into Water. Environ. Pollut..

[13] Marinello S., Lolli F., Gamberini R., Rimini B. (2020). A Second Life for Cigarette Butts? A Review of Recycling Solutions. J. Hazard. Mater..

[14] Dieng H., Rajasaygar S., Ahmad A.H., Rawi C.S.M., Ahmad H., Satho T., Miake F., Zuharah W.F., Fukumitsu Y., Saad A.R. (2014). Indirect Effects of Cigarette Butt Waste on the Dengue Vector Aedes Aegypti (Diptera: Culicidae). Acta Trop..

[15] Suárez-Rodríguez M., Garcia C.M. (2017). An Experimental Demonstration That House Finches Add Cigarette Butts in Response to Ectoparasites. J. Avian Biol..

[16] Dobaradaran S., Nabipour I., Saeedi R., Ostovar A., Khorsand M., Khajeahmadi N., Hayati R., Keshtkar M. (2017). Association of Metals (Cd, Fe, As, Ni, Cu, Zn and Mn) with Cigarette Butts in Northern Part of the Persian Gulf. Tob. Control.

[17] Kurmus H., Mohajerani A. (2020). The Toxicity and Valorization Options of Cigarette Butts. Waste Manag..

[18] Torkashvand J., Godini K., Norouzi S., Gholami M., Yeganeh M., Farzadkia M. (2021). Effect of Cigarette Butt on Concentration of Heavy Metals in Landfill Leachate: Health and Ecological Risk Assessment. J. Environ. Health Sci. Eng..

[19] Novotny T.E., Hardin S.N., Hovda L.R., Novotny D.J., McLean M.K., Khan S. (2011). Tobacco and Cigarette Butt Consumption in Humans and Animals. Tob. Control.

[20] Faisal M., Wu Z., Wang H., Hussain Z., Zhou Y., Wang H. (2022). Ecological and Health Risk Assessment of Dissolved Heavy Metals in the Urban Road Dust. Environ. Pollut. Bioavailab..

[21] Faisal M., Wu Z., Wang H., Lin X., Hussain Z., Azam M.I. (2022). Potential Heavy Metals Pollution Contribution from Wash-Off of Urban Road-Dust. Toxics.

[22] Faisal M., Wu Z., Wang H., Hussain Z., Shen C. (2021). Geochemical Mapping, Risk Assessment, and Source Identification of Heavy Metals in Road Dust Using Positive Matrix Factorization (PMF). Atmosphere.

[23] Faisal M., Wu Z., Wang H., Hussain Z., Azam M.I. (2021). Human Health Risk Assessment of Heavy Metals in the Urban Road Dust of Zhengzhou Metropolis, China. Atmosphere.

[24] Faisal M., You Z.-J., Akram M.Z., Ali S. (2023). Quantifying the Influence of Urban Road Surface Roughness on Heavy Metals Pollution in Road-Deposited Sediments Accumulation and Wash-Off. Water Sci. Technol..

[25] Faisal M., Wu Z., Wang H., Hussain Z., Azam M.I., Muzammil M. (2022). Assessment and Source Apportionment of Water-Soluble Heavy Metals in Road Dust of Zhengzhou, China. Environ. Sci. Pollut. Res..

[26] Bonanomi G., Maisto G., De Marco A., Cesarano G., Zotti M., Mazzei P., Libralato G., Staropoli A., Siciliano A., De Filippis F. (2020). The Fate of Cigarette Butts in Different Environments: Decay Rate, Chemical Changes and Ecotoxicity Revealed by a 5-Years Decomposition Experiment. Environ. Pollut..

[27] Booth D.J., Gribben P., Parkinson K. (2015). Impact of Cigarette Butt Leachate on Tidepool Snails. Mar. Pollut. Bull..

[28] Montalvão M.F., Sampaio L.L.G., Gomes H.H.F., Malafaia G. (2019). An Insight into the Cytotoxicity, Genotoxicity, and Mutagenicity of Smoked Cigarette Butt Leachate by Using Allium Cepa as Test System. Environ. Sci. Pollut. Res. Int..

[29] Caridi F., Sabbatini A., Birarda G., Costanzi E., De Giudici G., Galeazzi R., Medas D., Mobbili G., Ricciutelli M., Ruello M.L. (2020). Cigarette Butts, a Threat for Marine Environments: Lessons from Benthic Foraminifera (Protista). Mar. Environ. Res..

[30] Valiente R., Escobar F., Pearce J., Bilal U., Franco M., Sureda X. (2020). Estimating and Mapping Cigarette Butt Littering in Urban Environments: A GIS Approach. Environ. Res..

